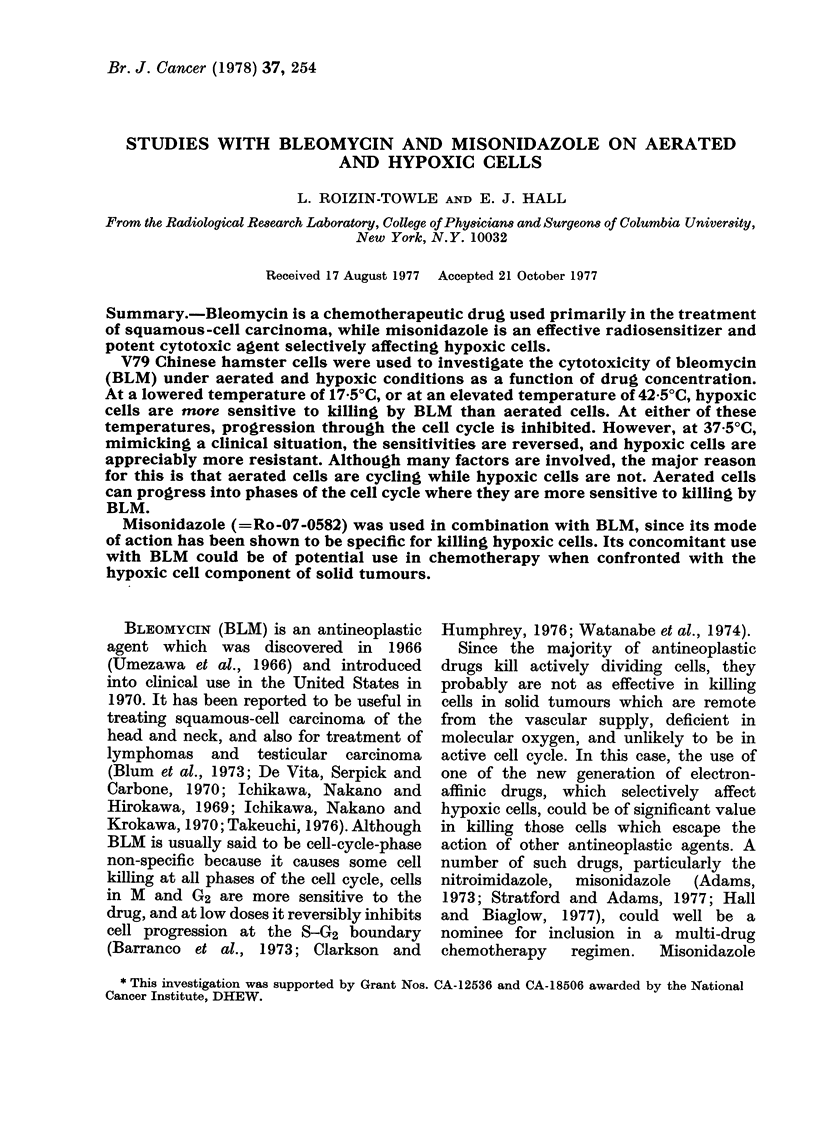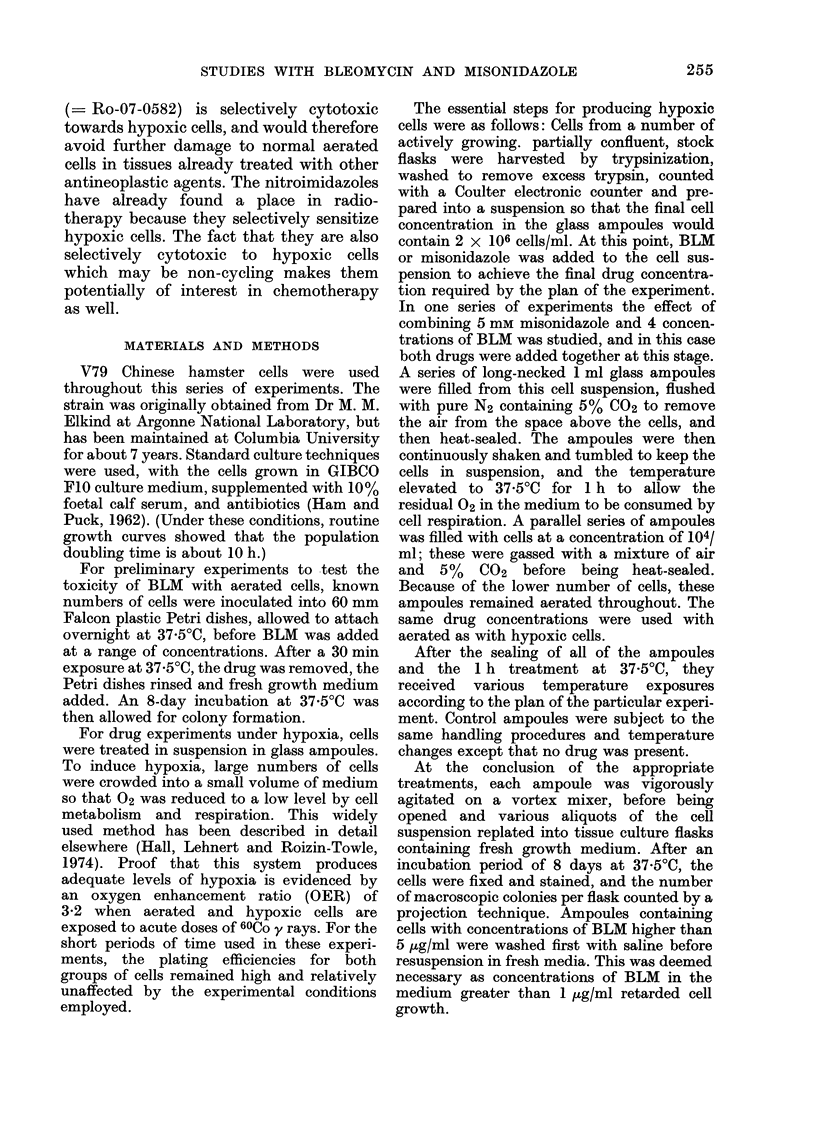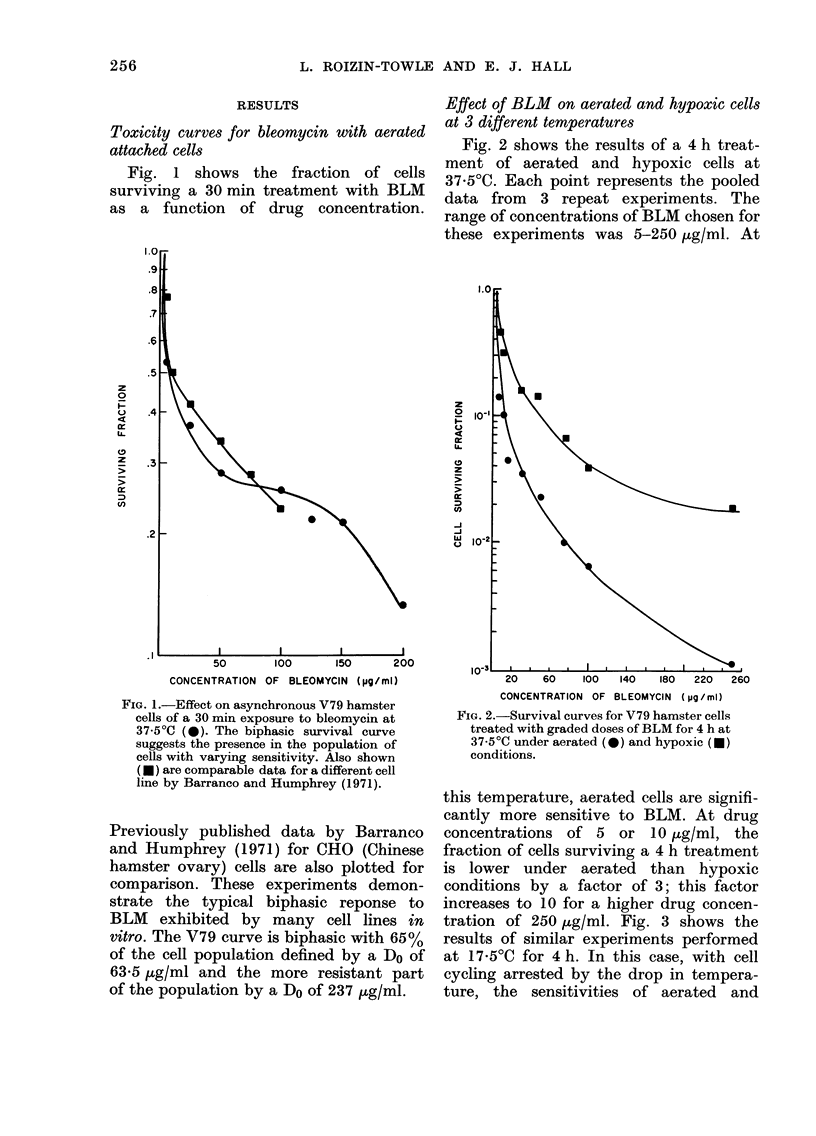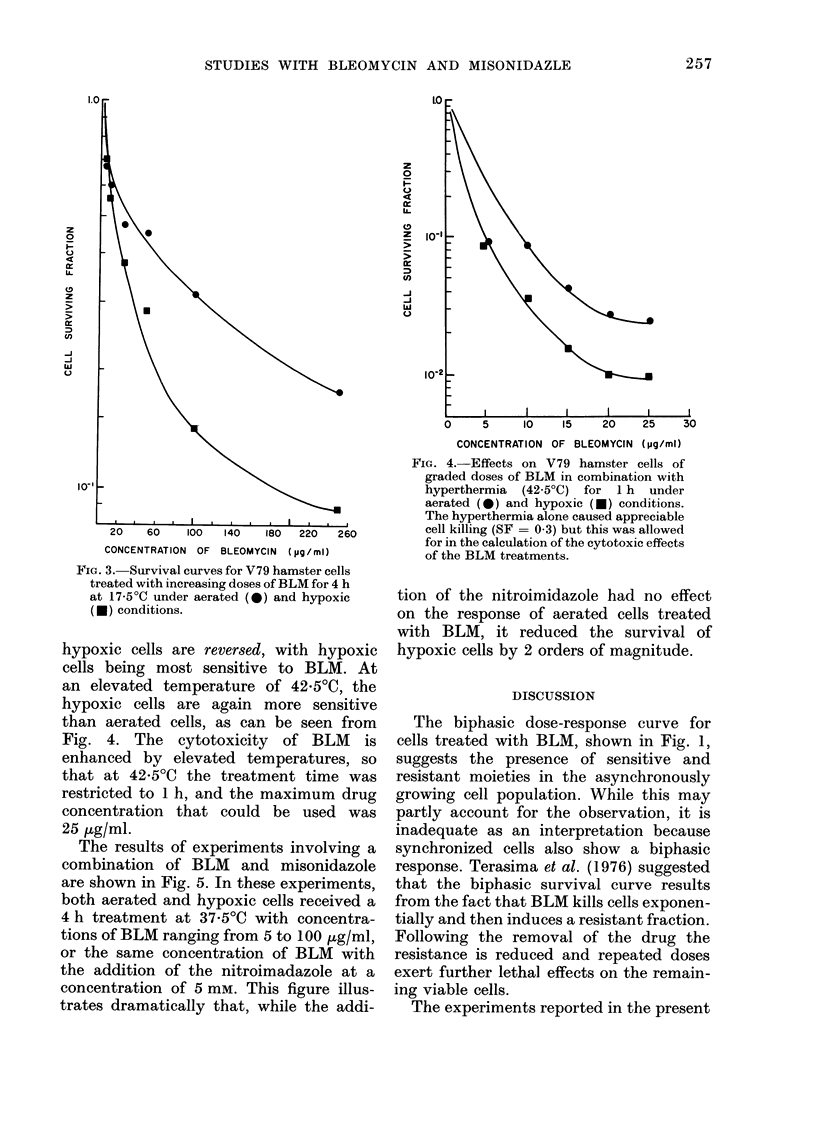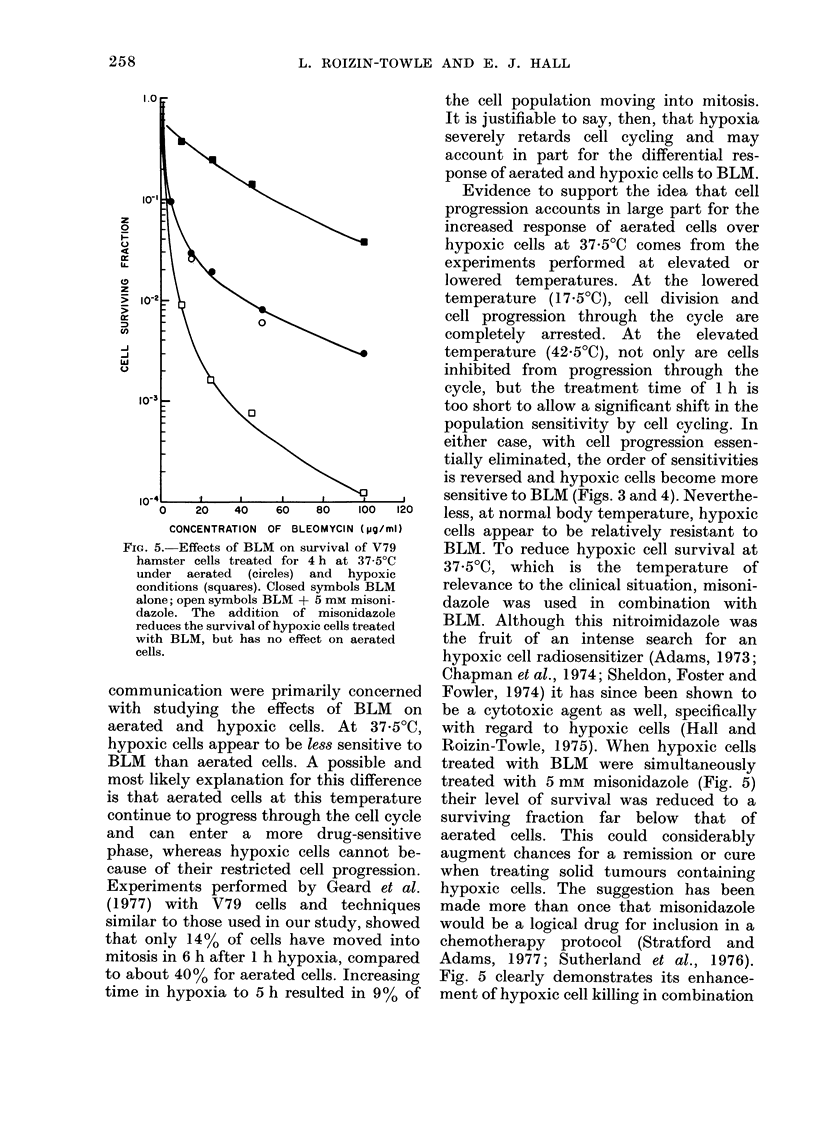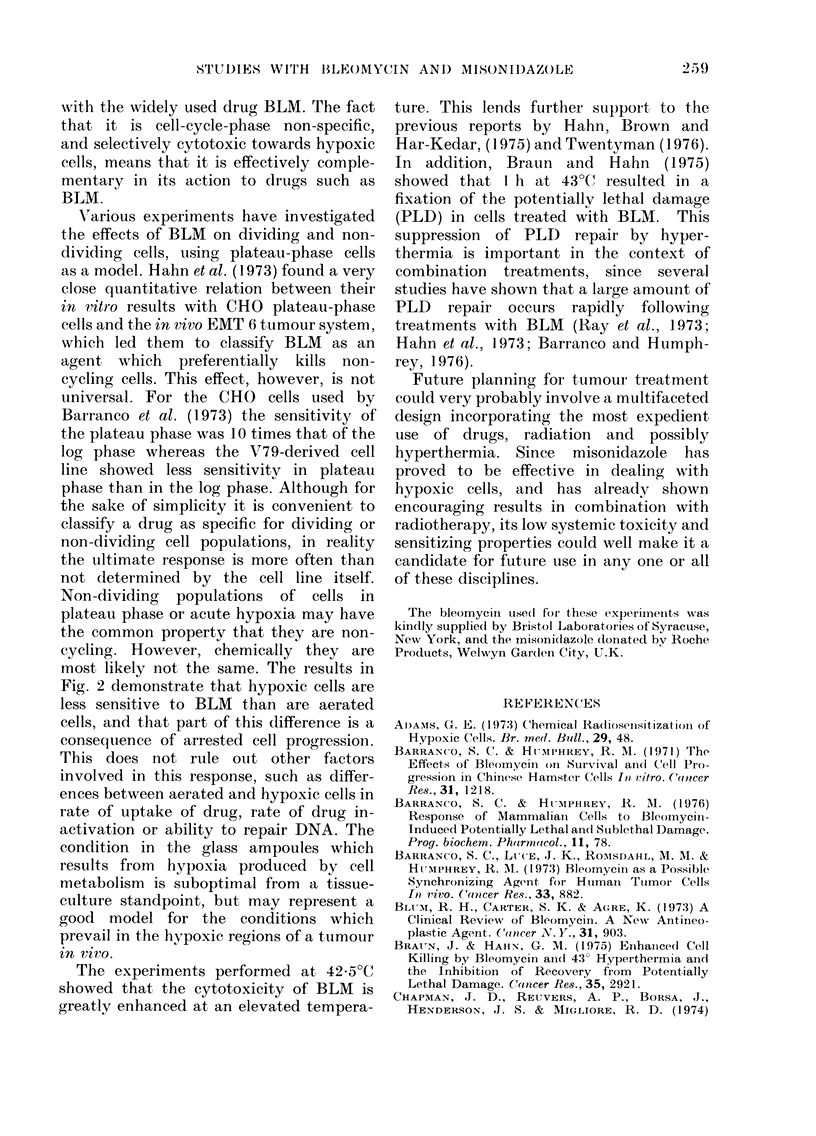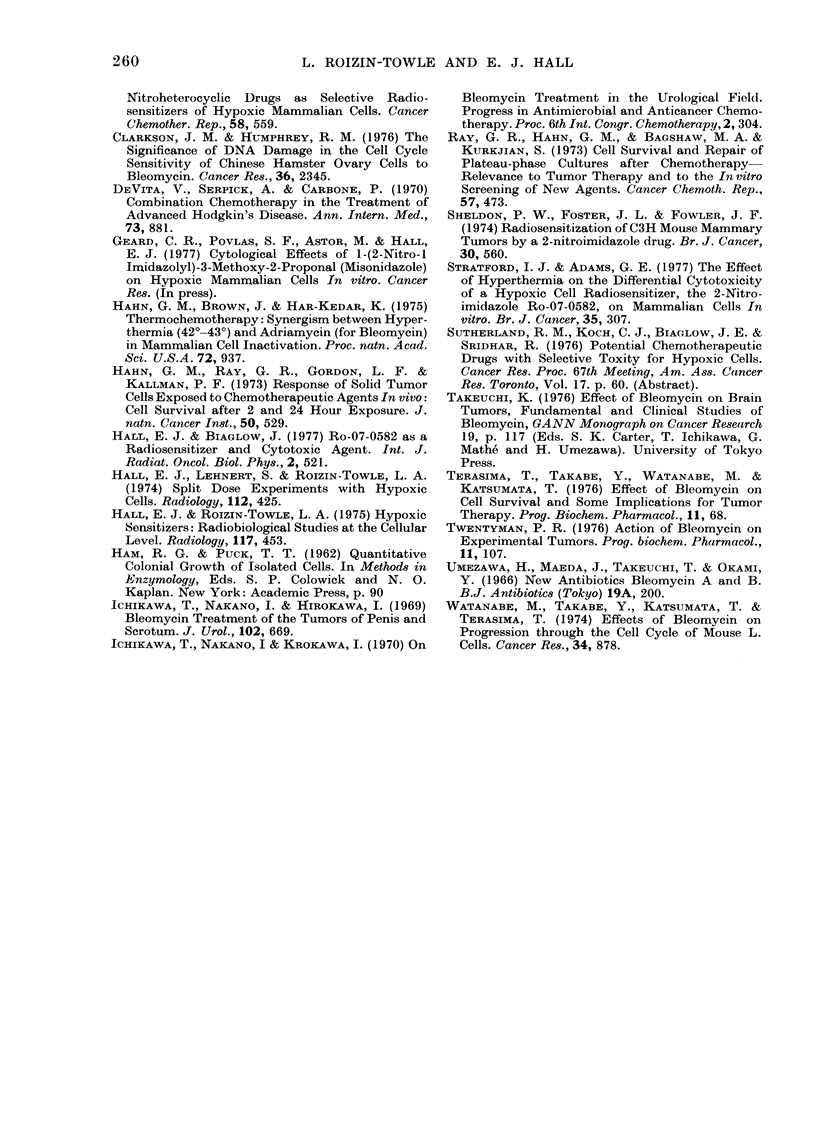# Studies with bleomycin and misonidazole on aerated and hypoxic cells.

**DOI:** 10.1038/bjc.1978.34

**Published:** 1978-02

**Authors:** L. Roizin-Towle, E. J. Hall

## Abstract

Bleomycin is a chemotherapuetic drug used primarily in the treatment of squamous-cell carcinoma, while misonidazole is an effective radiosensitizer and potent cytotoxic agent selectively affecting hypoxic cells. V79 Chinese hamster cells were used to investigate the cytotoxicity of bleomycin (BLM) under aerated and hypoxic conditions as a function of drug concentration. At a lowered temperature of 17.5 degrees C, or at an elevated temperature of 42.5 degrees C, hypoxic cells are more sensitive to killing by BLM than aerated cells. At either of these temperatures, progression through the cell cycle is inhibited. However, at 37.5 degrees C, mimicking a clinical situation, the sensitivies are reversed, and hypoxic cells are appreciably more resistant. Although many factors are involved, the major reason for this is that aerated cells are cycling while hypoxic cells are not. Aerated cells can progress into phases of the cell cycle where they are more sensitive to killing by BLM. Misoinidazole (=Ro-07-0582) was used in combination with BLM, since its mode of action has been shown to be psecific for killing hypoxic cells. It concomitant use with BLM could be of potential use in chemotherapy when confronted with the hypoxic cell component of solid tumours.


Br. J. Cancer (1978) 37, 254

### STUDIES WITH BLEOMYCIN AND MISONIDAZOLE ON AERATED AND HYPOXIC CELLS

L. ROIZIN-TOWLE AND E. J. HALL

*From the Radiological Research Laboratory, College of Physicians and Surgeons of Columbia University, New York, N.Y. 10032*

Received 17 August 1977 Accepted 21 October 1977

**Summary.—Bleomycin is a chemotherapeutic drug used primarily in the treatment of squamous-cell carcinoma, while misonidazole is an effective radiosensitizer and potent cytotoxic agent selectively affecting hypoxic cells.**

**V79 Chinese hamster cells were used to investigate the cytotoxicity of bleomycin (BLM) under aerated and hypoxic conditions as a function of drug concentration. At a lowered temperature of 17·5°C, or at an elevated temperature of 42·5°C, hypoxic cells are *more* sensitive to killing by BLM than aerated cells. At either of these temperatures, progression through the cell cycle is inhibited. However, at 37·5°C, mimicking a clinical situation, the sensitivities are reversed, and hypoxic cells are appreciably more resistant. Although many factors are involved, the major reason for this is that aerated cells are cycling while hypoxic cells are not. Aerated cells can progress into phases of the cell cycle where they are more sensitive to killing by BLM.**

**Misonidazole (=Ro-07-0582) was used in combination with BLM, since its mode of action has been shown to be specific for killing hypoxic cells. Its concomitant use with BLM could be of potential use in chemotherapy when confronted with the hypoxic cell component of solid tumours.**

BLEOMYCIN (BLM) is an antineoplastic agent which was discovered in 1966 (Umezawa *et al.*, 1966) and introduced into clinical use in the United States in 1970. It has been reported to be useful in treating squamous-cell carcinoma of the head and neck, and also for treatment of lymphomas and testicular carcinoma (Blum *et al.*, 1973; De Vita, Serpick and Carbone, 1970; Ichikawa, Nakano and Hirokawa, 1969; Ichikawa, Nakano and Krokawa, 1970; Takeuchi, 1976). Although BLM is usually said to be cell-cycle-phase non-specific because it causes some cell killing at all phases of the cell cycle, cells in M and $G_2$ are more sensitive to the drug, and at low doses it reversibly inhibits cell progression at the S–$G_2$ boundary (Barranco *et al.*, 1973; Clarkson and Humphrey, 1976; Watanabe *et al.*, 1974).

Since the majority of antineoplastic drugs kill actively dividing cells, they probably are not as effective in killing cells in solid tumours which are remote from the vascular supply, deficient in molecular oxygen, and unlikely to be in active cell cycle. In this case, the use of one of the new generation of electron-affinic drugs, which selectively affect hypoxic cells, could be of significant value in killing those cells which escape the action of other antineoplastic agents. A number of such drugs, particularly the nitroimidazole, misonidazole (Adams, 1973; Stratford and Adams, 1977; Hall and Biaglow, 1977), could well be a nominee for inclusion in a multi-drug chemotherapy regimen. Misonidazole

* This investigation was supported by Grant Nos. CA-12536 and CA-18506 awarded by the National Cancer Institute, DHEW.

(= Ro-07-0582) is selectively cytotoxic towards hypoxic cells, and would therefore avoid further damage to normal aerated cells in tissues already treated with other antineoplastic agents. The nitroimidazoles have already found a place in radiotherapy because they selectively sensitize hypoxic cells. The fact that they are also selectively cytotoxic to hypoxic cells which may be non-cycling makes them potentially of interest in chemotherapy as well.

### MATERIALS AND METHODS

V79 Chinese hamster cells were used throughout this series of experiments. The strain was originally obtained from Dr M. M. Elkind at Argonne National Laboratory, but has been maintained at Columbia University for about 7 years. Standard culture techniques were used, with the cells grown in GIBCO F10 culture medium, supplemented with 10% foetal calf serum, and antibiotics (Ham and Puck, 1962). (Under these conditions, routine growth curves showed that the population doubling time is about 10 h.)

For preliminary experiments to test the toxicity of BLM with aerated cells, known numbers of cells were inoculated into 60 mm Falcon plastic Petri dishes, allowed to attach overnight at 37·5°C, before BLM was added at a range of concentrations. After a 30 min exposure at 37·5°C, the drug was removed, the Petri dishes rinsed and fresh growth medium added. An 8-day incubation at 37·5°C was then allowed for colony formation.

For drug experiments under hypoxia, cells were treated in suspension in glass ampoules. To induce hypoxia, large numbers of cells were crowded into a small volume of medium so that $O_2$ was reduced to a low level by cell metabolism and respiration. This widely used method has been described in detail elsewhere (Hall, Lehnert and Roizin-Towle, 1974). Proof that this system produces adequate levels of hypoxia is evidenced by an oxygen enhancement ratio (OER) of 3·2 when aerated and hypoxic cells are exposed to acute doses of $^{60}Co$ $\gamma$ rays. For the short periods of time used in these experiments, the plating efficiencies for both groups of cells remained high and relatively unaffected by the experimental conditions employed.

The essential steps for producing hypoxic cells were as follows: Cells from a number of actively growing. partially confluent, stock flasks were harvested by trypsinization, washed to remove excess trypsin, counted with a Coulter electronic counter and prepared into a suspension so that the final cell concentration in the glass ampoules would contain $2 \times 10^6$ cells/ml. At this point, BLM or misonidazole was added to the cell suspension to achieve the final drug concentration required by the plan of the experiment. In one series of experiments the effect of combining 5 mM misonidazole and 4 concentrations of BLM was studied, and in this case both drugs were added together at this stage. A series of long-necked 1 ml glass ampoules were filled from this cell suspension, flushed with pure $N_2$ containing 5% $CO_2$ to remove the air from the space above the cells, and then heat-sealed. The ampoules were then continuously shaken and tumbled to keep the cells in suspension, and the temperature elevated to 37·5°C for 1 h to allow the residual $O_2$ in the medium to be consumed by cell respiration. A parallel series of ampoules was filled with cells at a concentration of $10^4$/ml; these were gassed with a mixture of air and 5% $CO_2$ before being heat-sealed. Because of the lower number of cells, these ampoules remained aerated throughout. The same drug concentrations were used with aerated as with hypoxic cells.

After the sealing of all of the ampoules and the 1 h treatment at 37·5°C, they received various temperature exposures according to the plan of the particular experiment. Control ampoules were subject to the same handling procedures and temperature changes except that no drug was present.

At the conclusion of the appropriate treatments, each ampoule was vigorously agitated on a vortex mixer, before being opened and various aliquots of the cell suspension replated into tissue culture flasks containing fresh growth medium. After an incubation period of 8 days at 37·5°C, the cells were fixed and stained, and the number of macroscopic colonies per flask counted by a projection technique. Ampoules containing cells with concentrations of BLM higher than 5 $\mu$g/ml were washed first with saline before resuspension in fresh media. This was deemed necessary as concentrations of BLM in the medium greater than 1 $\mu$g/ml retarded cell growth.

RESULTS

*Toxicity curves for bleomycin with aerated attached cells*

Fig. 1 shows the fraction of cells surviving a 30 min treatment with BLM as a function of drug concentration.

FIG. 1.—Effect on asynchronous V79 hamster cells of a 30 min exposure to bleomycin at 37·5°C (●). The biphasic survival curve suggests the presence in the population of cells with varying sensitivity. Also shown (■) are comparable data for a different cell line by Barranco and Humphrey (1971).

Previously published data by Barranco and Humphrey (1971) for CHO (Chinese hamster ovary) cells are also plotted for comparison. These experiments demonstrate the typical biphasic reponse to BLM exhibited by many cell lines *in vitro*. The V79 curve is biphasic with 65% of the cell population defined by a $D_0$ of 63·5 μg/ml and the more resistant part of the population by a $D_0$ of 237 μg/ml.

*Effect of BLM on aerated and hypoxic cells at 3 different temperatures*

Fig. 2 shows the results of a 4 h treatment of aerated and hypoxic cells at 37·5°C. Each point represents the pooled data from 3 repeat experiments. The range of concentrations of BLM chosen for these experiments was 5–250 μg/ml. At

FIG. 2.—Survival curves for V79 hamster cells treated with graded doses of BLM for 4 h at 37·5°C under aerated (●) and hypoxic (■) conditions.

this temperature, aerated cells are significantly more sensitive to BLM. At drug concentrations of 5 or 10 μg/ml, the fraction of cells surviving a 4 h treatment is lower under aerated than hypoxic conditions by a factor of 3; this factor increases to 10 for a higher drug concentration of 250 μg/ml. Fig. 3 shows the results of similar experiments performed at 17·5°C for 4 h. In this case, with cell cycling arrested by the drop in temperature, the sensitivities of aerated and

FIG. 3.—Survival curves for V79 hamster cells treated with increasing doses of BLM for 4 h at 17·5°C under aerated (●) and hypoxic (■) conditions.

FIG. 4.—Effects on V79 hamster cells of graded doses of BLM in combination with hyperthermia (42·5°C) for 1 h under aerated (●) and hypoxic (■) conditions. The hyperthermia alone caused appreciable cell killing (SF = 0·3) but this was allowed for in the calculation of the cytotoxic effects of the BLM treatments.

hypoxic cells are *reversed*, with hypoxic cells being most sensitive to BLM. At an elevated temperature of 42·5°C, the hypoxic cells are again more sensitive than aerated cells, as can be seen from Fig. 4. The cytotoxicity of BLM is enhanced by elevated temperatures, so that at 42·5°C the treatment time was restricted to 1 h, and the maximum drug concentration that could be used was 25 μg/ml.

The results of experiments involving a combination of BLM and misonidazole are shown in Fig. 5. In these experiments, both aerated and hypoxic cells received a 4 h treatment at 37·5°C with concentrations of BLM ranging from 5 to 100 μg/ml, or the same concentration of BLM with the addition of the nitroimadazole at a concentration of 5 mM. This figure illustrates dramatically that, while the addition of the nitroimidazole had no effect on the response of aerated cells treated with BLM, it reduced the survival of hypoxic cells by 2 orders of magnitude.

### DISCUSSION

The biphasic dose-response curve for cells treated with BLM, shown in Fig. 1, suggests the presence of sensitive and resistant moieties in the asynchronously growing cell population. While this may partly account for the observation, it is inadequate as an interpretation because synchronized cells also show a biphasic response. Terasima *et al.* (1976) suggested that the biphasic survival curve results from the fact that BLM kills cells exponentially and then induces a resistant fraction. Following the removal of the drug the resistance is reduced and repeated doses exert further lethal effects on the remaining viable cells.

The experiments reported in the present

FIG. 5.—Effects of BLM on survival of V79 hamster cells treated for 4 h at 37·5°C under aerated (circles) and hypoxic conditions (squares). Closed symbols BLM alone; open symbols BLM + 5 mM misonidazole. The addition of misonidazole reduces the survival of hypoxic cells treated with BLM, but has no effect on aerated cells.

communication were primarily concerned with studying the effects of BLM on aerated and hypoxic cells. At 37·5°C, hypoxic cells appear to be *less* sensitive to BLM than aerated cells. A possible and most likely explanation for this difference is that aerated cells at this temperature continue to progress through the cell cycle and can enter a more drug-sensitive phase, whereas hypoxic cells cannot because of their restricted cell progression. Experiments performed by Geard *et al.* (1977) with V79 cells and techniques similar to those used in our study, showed that only 14% of cells have moved into mitosis in 6 h after 1 h hypoxia, compared to about 40% for aerated cells. Increasing time in hypoxia to 5 h resulted in 9% of the cell population moving into mitosis. It is justifiable to say, then, that hypoxia severely retards cell cycling and may account in part for the differential response of aerated and hypoxic cells to BLM.

Evidence to support the idea that cell progression accounts in large part for the increased response of aerated cells over hypoxic cells at 37·5°C comes from the experiments performed at elevated or lowered temperatures. At the lowered temperature (17·5°C), cell division and cell progression through the cycle are completely arrested. At the elevated temperature (42·5°C), not only are cells inhibited from progression through the cycle, but the treatment time of 1 h is too short to allow a significant shift in the population sensitivity by cell cycling. In either case, with cell progression essentially eliminated, the order of sensitivities is reversed and hypoxic cells become more sensitive to BLM (Figs. 3 and 4). Nevertheless, at normal body temperature, hypoxic cells appear to be relatively resistant to BLM. To reduce hypoxic cell survival at 37·5°C, which is the temperature of relevance to the clinical situation, misonidazole was used in combination with BLM. Although this nitroimidazole was the fruit of an intense search for an hypoxic cell radiosensitizer (Adams, 1973; Chapman *et al.*, 1974; Sheldon, Foster and Fowler, 1974) it has since been shown to be a cytotoxic agent as well, specifically with regard to hypoxic cells (Hall and Roizin-Towle, 1975). When hypoxic cells treated with BLM were simultaneously treated with 5 mM misonidazole (Fig. 5) their level of survival was reduced to a surviving fraction far below that of aerated cells. This could considerably augment chances for a remission or cure when treating solid tumours containing hypoxic cells. The suggestion has been made more than once that misonidazole would be a logical drug for inclusion in a chemotherapy protocol (Stratford and Adams, 1977; Sutherland *et al.*, 1976). Fig. 5 clearly demonstrates its enhancement of hypoxic cell killing in combination with the widely used drug BLM. The fact that it is cell-cycle-phase non-specific, and selectively cytotoxic towards hypoxic cells, means that it is effectively complementary in its action to drugs such as BLM.

Various experiments have investigated the effects of BLM on dividing and non-dividing cells, using plateau-phase cells as a model. Hahn *et al.* (1973) found a very close quantitative relation between their *in vitro* results with CHO plateau-phase cells and the *in vivo* EMT 6 tumour system, which led them to classify BLM as an agent which preferentially kills non-cycling cells. This effect, however, is not universal. For the CHO cells used by Barranco *et al.* (1973) the sensitivity of the plateau phase was 10 times that of the log phase whereas the V79-derived cell line showed less sensitivity in plateau phase than in the log phase. Although for the sake of simplicity it is convenient to classify a drug as specific for dividing or non-dividing cell populations, in reality the ultimate response is more often than not determined by the cell line itself. Non-dividing populations of cells in plateau phase or acute hypoxia may have the common property that they are non-cycling. However, chemically they are most likely not the same. The results in Fig. 2 demonstrate that hypoxic cells are less sensitive to BLM than are aerated cells, and that part of this difference is a consequence of arrested cell progression. This does not rule out other factors involved in this response, such as differences between aerated and hypoxic cells in rate of uptake of drug, rate of drug inactivation or ability to repair DNA. The condition in the glass ampoules which results from hypoxia produced by cell metabolism is suboptimal from a tissue-culture standpoint, but may represent a good model for the conditions which prevail in the hypoxic regions of a tumour *in vivo*.

The experiments performed at 42·5°C showed that the cytotoxicity of BLM is greatly enhanced at an elevated temperature. This lends further support to the previous reports by Hahn, Brown and Har-Kedar, (1975) and Twentyman (1976). In addition, Braun and Hahn (1975) showed that 1 h at 43°C resulted in a fixation of the potentially lethal damage (PLD) in cells treated with BLM. This suppression of PLD repair by hyperthermia is important in the context of combination treatments, since several studies have shown that a large amount of PLD repair occurs rapidly following treatments with BLM (Ray *et al.*, 1973; Hahn *et al.*, 1973; Barranco and Humphrey, 1976).

Future planning for tumour treatment could very probably involve a multifaceted design incorporating the most expedient use of drugs, radiation and possibly hyperthermia. Since misonidazole has proved to be effective in dealing with hypoxic cells, and has already shown encouraging results in combination with radiotherapy, its low systemic toxicity and sensitizing properties could well make it a candidate for future use in any one or all of these disciplines.

The bleomycin used for these experiments was kindly supplied by Bristol Laboratories of Syracuse, New York, and the misonidazole donated by Roche Products, Welwyn Garden City, U.K.

### REFERENCES

ADAMS, G. E. (1973) Chemical Radiosensitization of Hypoxic Cells. *Br. med. Bull.*, **29**, 48.

BARRANCO, S. C. & HUMPHREY, R. M. (1971) The Effects of Bleomycin on Survival and Cell Progression in Chinese Hamster Cells *In vitro*. *Cancer Res.*, **31**, 1218.

BARRANCO, S. C. & HUMPHREY, R. M. (1976) Response of Mammalian Cells to Bleomycin-Induced Potentially Lethal and Sublethal Damage. *Prog. biochem. Pharmacol.*, **11**, 78.

BARRANCO, S. C., LUCE, J. K., ROMSDAHL, M. M. & HUMPHREY, R. M. (1973) Bleomycin as a Possible Synchronizing Agent for Human Tumor Cells *In vivo*. *Cancer Res.*, **33**, 882.

BLUM, R. H., CARTER, S. K. & AGRE, K. (1973) A Clinical Review of Bleomycin. A New Antineoplastic Agent. *Cancer N.Y.*, **31**, 903.

BRAUN, J. & HAHN, G. M. (1975) Enhanced Cell Killing by Bleomycin and 43° Hyperthermia and the Inhibition of Recovery from Potentially Lethal Damage. *Cancer Res.*, **35**, 2921.

CHAPMAN, J. D., REUVERS, A. P., BORSA, J., HENDERSON, J. S. & MIGLIORE, R. D. (1974)

Nitroheterocyclic Drugs as Selective Radiosensitizers of Hypoxic Mammalian Cells. *Cancer Chemother. Rep.*, **58**, 559.

CLARKSON, J. M. & HUMPHREY, R. M. (1976) The Significance of DNA Damage in the Cell Cycle Sensitivity of Chinese Hamster Ovary Cells to Bleomycin. *Cancer Res.*, **36**, 2345.

DEVITA, V., SERPICK, A. & CARBONE, P. (1970) Combination Chemotherapy in the Treatment of Advanced Hodgkin's Disease. *Ann. Intern. Med.*, **73**, 881.

GEARD, C. R., POVLAS, S. F., ASTOR, M. & HALL, E. J. (1977) Cytological Effects of 1-(2-Nitro-1 Imidazolyl)-3-Methoxy-2-Proponal (Misonidazole) on Hypoxic Mammalian Cells *In vitro*. *Cancer Res.* (In press).

HAHN, G. M., BROWN, J. & HAR-KEDAR, K. (1975) Thermochemotherapy: Synergism between Hyperthermia (42°–43°) and Adriamycin (for Bleomycin) in Mammalian Cell Inactivation. *Proc. natn. Acad. Sci. U.S.A.* **72**, 937.

HAHN, G. M., RAY, G. R., GORDON, L. F. & KALLMAN, P. F. (1973) Response of Solid Tumor Cells Exposed to Chemotherapeutic Agents *In vivo*: Cell Survival after 2 and 24 Hour Exposure. *J. natn. Cancer Inst.*, **50**, 529.

HALL, E. J. & BIAGLOW, J. (1977) Ro-07-0582 as a Radiosensitizer and Cytotoxic Agent. *Int. J. Radiat. Oncol. Biol. Phys.*, **2**, 521.

HALL, E. J., LEHNERT, S. & ROIZIN-TOWLE, L. A. (1974) Split Dose Experiments with Hypoxic Cells. *Radiology*, **112**, 425.

HALL, E. J. & ROIZIN-TOWLE, L. A. (1975) Hypoxic Sensitizers: Radiobiological Studies at the Cellular Level. *Radiology*, **117**, 453.

HAM, R. G. & PUCK, T. T. (1962) Quantitative Colonial Growth of Isolated Cells. In *Methods in Enzymology*, Eds. S. P. Colowick and N. O. Kaplan. New York: Academic Press, p. 90

ICHIKAWA, T., NAKANO, I. & HIROKAWA, I. (1969) Bleomycin Treatment of the Tumors of Penis and Scrotum. *J. Urol.*, **102**, 669.

ICHIKAWA, T., NAKANO, I & KROKAWA, I. (1970) On Bleomycin Treatment in the Urological Field. Progress in Antimicrobial and Anticancer Chemotherapy. *Proc. 6th Int. Congr. Chemotherapy*, **2**, 304.

RAY, G. R., HAHN, G. M., & BAGSHAW, M. A. & KURKJIAN, S. (1973) Cell Survival and Repair of Plateau-phase Cultures after Chemotherapy—Relevance to Tumor Therapy and to the *In vitro* Screening of New Agents. *Cancer Chemoth. Rep.*, **57**, 473.

SHELDON, P. W., FOSTER, J. L. & FOWLER, J. F. (1974) Radiosensitization of C3H Mouse Mammary Tumors by a 2-nitroimidazole drug. *Br. J. Cancer*, **30**, 560.

STRATFORD, I. J. & ADAMS, G. E. (1977) The Effect of Hyperthermia on the Differential Cytotoxicity of a Hypoxic Cell Radiosensitizer, the 2-Nitroimidazole Ro-07-0582, on Mammalian Cells *In vitro*. *Br. J. Cancer*, **35**, 307.

SUTHERLAND, R. M., KOCH, C. J., BIAGLOW, J. E. & SRIDHAR, R. (1976) Potential Chemotherapeutic Drugs with Selective Toxity for Hypoxic Cells. *Cancer Res. Proc. 67th Meeting, Am. Ass. Cancer Res. Toronto*, Vol. 17. p. 60. (Abstract).

TAKEUCHI, K. (1976) Effect of Bleomycin on Brain Tumors, Fundamental and Clinical Studies of Bleomycin, *GANN Monograph on Cancer Research* 19, p. 117 (Eds. S. K. Carter, T. Ichikawa, G. Mathé and H. Umezawa). University of Tokyo Press.

TERASIMA, T., TAKABE, Y., WATANABE, M. & KATSUMATA, T. (1976) Effect of Bleomycin on Cell Survival and Some Implications for Tumor Therapy. *Prog. Biochem. Pharmacol.*, **11**, 68.

TWENTYMAN, P. R. (1976) Action of Bleomycin on Experimental Tumors. *Prog. biochem. Pharmacol.*, **11**, 107.

UMEZAWA, H., MAEDA, J., TAKEUCHI, T. & OKAMI, Y. (1966) New Antibiotics Bleomycin A and B. *B.J. Antibiotics (Tokyo)* **19A**, 200.

WATANABE, M., TAKABE, Y., KATSUMATA, T. & TERASIMA, T. (1974) Effects of Bleomycin on Progression through the Cell Cycle of Mouse L. Cells. *Cancer Res.*, **34**, 878.